# Scaling and Interactions of Linear and Ring Polymer Brushes via DPD Simulations

**DOI:** 10.3390/polym11030541

**Published:** 2019-03-22

**Authors:** Martin Jehser, Gerhard Zifferer, Christos N. Likos

**Affiliations:** 1 Faculty of Chemistry, University of Vienna, Währinger Straße 42, A-1090 Vienna, Austria; 2 Faculty of Physics, University of Vienna, Boltzmanngasse 5, A-1090 Vienna, Austria; christos.likos@univie.ac.at

**Keywords:** DPD, polymer brush, polymer rings, computer simulation, scaling theory, effective interactions

## Abstract

Single and double layers of polymer coated surfaces are investigated by means of Dissipative Particle Dynamics (DPD), focusing on the difference between grafted ring and linear chains. Several different surface coverages σ, as well as chain lengths *N* and brush separations *D*, are analyzed for athermal, i.e., good solvent, conditions. The size in the form of the radius of gyration $R_{g}$, the shape as asphericity $\delta^{*}$, and orientation $\beta^{*}$, as well as density profiles as functions of distance from grafting plane ρ(z), are studied. The effect of an added bond repulsion potential to suppress bond crossing in DPD is analyzed. Scaling laws of $R_{g}$ and its components $R_{g⊥}$ and $R_{g∥}$ are investigated. We find $R_{g}\propto N^{\nu },\nu =0.588$ for surface coverages below the overlap surface concentration $\sigma_{*}$. For $\sigma >\sigma_{*}$ we find $R_{g⊥}\propto N^{\nu_{⊥}},\nu_{⊥}≅1$ and $R_{g∥}\propto N^{\nu_{∥}},\nu_{∥}=1/2$ of ring brushes with the standard DPD model and $\nu_{∥}≅2/5$ with added bond repulsion. The σ dependence of the radius of gyration was found to be $R_{g}\propto \sigma^{\mu }$ with μ=1/3 for surface coverages grater than $\sigma_{*}$. The perpendicular component $R_{g⊥}$ scales independent of the bond repulsion potential as $R_{g⊥}\propto \sigma^{\mu_{⊥}},\mu_{⊥}=1/3$, whereas the scaling of the parallel component exhibits a topological repulsion dependence $R_{g∥}\propto \sigma^{\mu_{∥}},\mu_{∥}=-1/12$ for standard DPD and $\mu_{∥}=-1/6$ for bond repulsion.

## 1. Introduction

The process of grafting polymers to a surface paves the way into a versatile and interesting field of technological and industrial, as well as academic, uses. Oil recovery, friction, lubrication adhesion and wetting properties, colloidal stabilization, modification of surface chemistry, biocompatibility, protective coatings, and microfluidic devices are only some of the manifold applications conceivable with polymer coated surfaces. Due to this broad field of application it is no wonder that, during the last few decades, polymer brushes have been a subject of intensive study by experiment [1,2,3,4,5,6,7,8,9,10,11,12,13], theory [14,15,16,17,18,19,20,21,22,23,24,25,26,27,28,29,30,31], and simulation [32,33,34,35,36,37,38,39,40,41,42,43,44,45,46,47,48,49,50,51,52]. Prominent reference models for the behavior of dense planar polymer brushes are the Alexander-deGennes brush [14,15], as well as the celebrated parabolic polymer brush emerging from the self-consistent field approach by Milner, Witten and Cates [17].

Most of the simulation work is focused on grafted linear polymer chains in various conditions. Relatively little in known about ring polymer brushes [50]. The aim of the current contribution is to expand the field of ring brush investigations by employing the tool of Dissipative Particle Dynamics Simulations (DPD). We present simulation results for polymer brushes of linear and ring polymers grafted onto a flat substrate, focusing on the differences between these two polymer geometries. We investigate differences in statistical and structural properties such as the radius of gyration $R_{g}$, shape in the form of the asphericity $\delta^{*}$, and monomer density profiles as a function of distance from the surface ρ(z). We take a closer look at scaling dependence of $R_{g}$ and its parallel $R_{g∥}$ and perpendicular $R_{g⊥}$ components as functions of effective chain length *N* and surface coverage σ. By incorporating the bond repulsion potential by Sirk et al. [53] we strongly reduce the possibility of bond crossing and compare the results to standard DPD model. In a next step we investigate a combined system of two opposing brushes, a slit confinement, bringing them closer together until we reach a compressed bilayer brush. Here we study the aforementioned quantities in dependence of the brush distance *D* and we take a closer look at the interaction force $F_{int}$ and the interpenetration in the form of the integral of overlap $I_{ov}$ of the two brushes.

In Section 2 we present the Dissipative Particle Dynamics Model method and our Model of the polymer brush, as well as our implementation of the segmental repulsion model. In Section 3 we go into the details of the simulation and we present and discuss our findings in Section 4, with the focus on a single brush in Section 4.1 and onto the bilayer brush system in Section 4.2.

## 2. Model and Methods

### 2.1. The General DPD Model

Dissipative Particle Dynamics (DPD), in its original form, was developed by Hoogerbrugge and Koelman in 1992 [54] and improved upon by Español and Warren in 1995 [55] as a mesoscopic simulation tool for complex fluids. DPD is in essence a coarse-grained molecular dynamics simulation including dissipative and stochastic interactions, where each point particle, henceforth called a bead, represents a group of atoms or a volume of fluid. These beads interact via a purely repulsive conservative force $F_{ij}^{C}$, a dissipative force counteracting velocity differences between particles $F_{ij}^{D}$, and a stochastic force directed along the connection line between particle centers $F_{ij}^{R}$, each being pairwise additive. This approach was based on the description of the Brownian motion of particles in a potential by the Langevin equation of motion, but contrary to Brownian or Langevin dynamics is momentum conserving. The original DPD model is described by

(1) $$\frac{dr_{i}}{dt}=v_{i},$$

(2) $$m_{i}\frac{dv_{i}}{dt}=F_{i},$$

where $r_{i}$, $v_{i}$, and $m_{i}$ are the position, velocity, and mass of particle *i*, respectively. The total force $F_{i}$ acting on each bead is:

(3) $$F_{i}=\sum_{i\neq j}\left(F_{ij}^{C}+F_{ij}^{D}+F_{ij}^{R}\right).$$

The conservative force is given by:

(4) $$F_{ij}^{C}\left(r\right)=\left\{\begin{array}{cc} a_{ij}\left(1-\frac{r_{ij}}{r_{c}}\right){\hat{r}}_{ij} & \mathrm{if}\;r_{ij}\leq r_{c}\\ 0 & \mathrm{if}\;r_{ij}>r_{c} \end{array}\right..$$

Accordingly $F_{ij}^{C}\left(r\right)$ is a scalar non-negative (repulsive) function determining the form of the conservative interaction, $a_{ij}$ is the parameter of maximum repulsion between beads *i* and *j*, $r_{c}$ is the cut-off distance, $r_{ij}=r_{i}-r_{j}$ the distance between particles, $r_{ij}=∥r_{ij}∥$ is its magnitude, and ${\hat{r}}_{ij}=r_{ij}/r_{ij}$ is the unit vector from bead *j* to *i*.

Based on extensive work of Español and Warren in 1995 [55] it is known that the stochastic $F_{ij}^{R}$ and dissipative $F_{ij}^{D}$ forces need to be coupled together through a fluctuation-dissipation relation to ensure that the system in thermodynamic equilibrium stays in the canonical (NVT) ensemble. This leads to

(5) $$\begin{array}{c} \begin{array}{cc} F_{ij}^{D} & =-\gamma \omega^{D}\left(r_{ij}\right)\left({\hat{r}}_{ij}\cdot v_{ij}\right){\hat{r}}_{ij},\\ F_{ij}^{R} & =-\sigma_{DPD}\omega^{R}\left(r_{ij}\right)\zeta_{ij}{\hat{r}}_{ij}, \end{array} \end{array}$$

with the condition

(6) $$\sigma_{DPD}^{2}=2\gamma k_{B}T,$$

where γ and $\sigma_{DPD}$ are parameters determining the strength of the dissipative and stochastic interaction, $k_{B}$ is Boltzmann’s constant, *T* the temperature, and $v_{ij}=v_{i}-v_{j}$ is the difference in velocity of the beads *i* and *j*. Moreover, $\zeta_{ij}$ is a symmetric Gaussian random variable with zero mean and unit variance, which is independent for different pairs of particles and at different times. The symmetry relation $\zeta_{ij}=\zeta_{ji}$ is enforced to satisfy momentum conservation of the stochastic force, whereas $\omega^{D}\left(r_{ij}\right)$ and $\omega^{R}\left(r_{ij}\right)$ are weight functions satisfying

(7) $$\omega^{D}\left(r_{ij}\right)={\left[\omega^{R}\left(r_{ij}\right)\right]}^{2}.$$

For simplicity, the weight functions are selected to be similar in form to the conservative force $F^{C}\left(r_{ij}\right)$ Equation (4), that is

(8) $$\omega^{D}\left(r_{ij}\right)={\left[\omega^{R}\left(r_{ij}\right)\right]}^{2}=\left\{\begin{array}{cc} {\left(1-\frac{r_{ij}}{r_{c}}\right)}^{2} & \mathrm{if}\;r_{ij}\leq r_{c}\\ 0 & \mathrm{if}\;r_{ij}>r_{c} \end{array}\right..$$

Due to the fact that all interactions are pairwise additive, they obey Newton’s third law and all forces depend only on relative positions $r_{ij}$ and velocities $v_{ij}$, linear and angular momentum is conserved, and the model is Galilean-invariant. These conditions make DPD into a consistent hydrodynamic model particularly interesting for the study of mesoscopic soft matter systems with length and time scales ranging from $10-10^{4}$ nm and $1-10^{6}$ ns.

Time integration is performed with a modified version of the velocity-Verlet algorithm [56]:

(9) $$\begin{array}{c} \begin{array}{cc} r_{i}\left(t+\Delta t\right) & =r_{i}\left(t\right)+\Delta tv_{i}\left(t\right)+\frac{1}{2}{\left(\Delta t\right)}^{2}F_{i}\left(t\right),\\ {\tilde{v}}_{i}\left(t+\Delta t\right) & =v_{i}\left(t\right)+\lambda \Delta tF_{i}\left(t\right),\\ F_{i}\left(t+\Delta t\right) & =F_{i}\left(r_{i}\left(t+\Delta t\right),{\tilde{v}}_{i}\left(t+\Delta t\right)\right),\\ v_{i}\left(t+\Delta t\right) & =v_{i}\left(t\right)+\frac{1}{2}\Delta t\left(F_{i}\left(t\right)+F_{i}\left(t+\Delta t\right)\right). \end{array} \end{array}$$

Here, λ is an empirical variable factor influencing the stability of the thermostat. The standard velocity-Verlet algorithm would be recovered for λ=1/2 for a force term independent of the velocity. Because the dissipative force is dependent on the velocity however, a prediction of the new velocity $\tilde{v}$ is needed and the corrected velocity is evaluated in the last step of the integration. Forces are updated once per iteration after the second step leading to virtually no increase in computational cost.

### 2.2. The Brush Model

For the simulation of the polymer, a DPD bead can either be a solvent particle or a monomer. Any two consecutive monomer DPD beads *i* and *j* forming a bond are connected via a spring force with the spring constant $b_{ij}$, changing Equation (4) to

(10) $$F_{ij}^{C}\left(r\right)=\left\{\begin{array}{cc} a_{ij}\left(1-\frac{r_{ij}}{r_{c}}\right){\hat{r}}_{ij}-b_{ij}r_{ij} & \mathrm{if}\;r_{ij}\leq r_{c}\\ -b_{ij}r_{ij} & \mathrm{if}\;r_{ij}>r_{c} \end{array}\right..$$

There are no angle potentials in the DPD chain, leading to a freely jointed polymer so that the bead comprises of at least one Kuhn segment. Due to the softness of the repulsive interaction and the relative large time step of the DPD simulation, bond crossing cannot be prevented without the implementation of further interactions or the shift to a hard repulsive interaction potential. See Section 2.3 for more details on the bond repulsion potential mSRP [53].

The polymers are grafted on walls parallel to the xy-plane. Periodic boundary conditions in the *x* and *y* directions and a repulsive soft wall in the *z* direction, with repulsion parameter $d_{i}$ as shown in Equation (11), capping the top and bottom of the simulation box, are employed. To link brush polymers to the surface, an attractive spring with the spiring constant $c_{i}$ was added to the end monomer or monomers for linear or ring conformations respectively, effectively adsorbing the end monomers to the surface. Equation (11) summarizes the additional surface force $F_{i}^{surf}$ added to DPD bead *i*, with *D* being the distance between the walls, where $c_{i}=0$ for every bead except the anchored ends,

(11) $$F_{i}^{surf}=\left\{\begin{array}{cc} -d_{i}z_{i}-c_{i}z_{i} & \mathrm{if}\;z_{i}<0\\ -c_{i}z_{i} & \mathrm{if}\;0\leq z_{i}\leq D\\ -d_{i}\left(z_{i}-D\right)-c_{i}z_{i} & \mathrm{if}\;z_{i}>D \end{array}\right..$$

Reduced variables are used throughout this paper. In DPD energy is measured in units of $k_{B}T$, length in units of the force cutoff radius $r_{c}$, and mass in units of *m*, the mass of a single DPD bead. In the current contribution $k_{B}T$, *m* and $r_{c}$ are set to unity. The noise amplitude $\sigma_{DPD}$ is set equal to 3, with Equation (6) leading to γ=4.5. The number density $\rho_{DPD}=3$ and the time integration parameter λ=0.65 and timestep Δt=0.04 were chosen according to a study by Groot and Warren [56]. Also shown within reference [56], through a series of equilibrium simulations, is that for sufficiently high number densities $\left(\rho_{DPD}>2\right)$, a good approximation for the DPD equation of state is given by

(12) $$p=\rho_{DPD}k_{B}T+\alpha a\rho_{DPD}^{2}\;\;\left(\alpha =0.101\pm 0.001\right).$$

This leads to the dimensionless compressibility

(13) $$\kappa^{-1}\approx 1+0.2a\rho_{DPD}/k_{B}T$$

for the DPD model of a given conservative interaction strength $a=a_{ij}$ for all i,j pairs. According to the discussed reference, the compressibility of water at room temperature (300K) is $\kappa^{-1}=15.9835\approx 16$. For the given number density $\rho_{DPD}=3$ in the current work, Equation (13) in combination with the compressibility of water, leads to an conservative interaction parameter of $a=25k_{B}T$. To simulate an athermal polymer solution the interaction of all DPD bead species (e.g., solvent beads and polymer beads) is set equally to $a=25k_{B}T$ as well. The spring constants connecting the monomer beads to each other is set to $b_{ij}=4$ and to the surface for the fist (tails) and first and last (rings) monomer is $c_{i}\;=\;24$. The value of the soft wall repulsion parameter is $d_{i}=100$ for all beads, i.e., solvent or monomer.

### 2.3. Midpoint Bond Repulsion

The biggest advantage of DPD sometimes is also its drawback. Due to the soft interaction potentials and the large time steps employed in this method, the unphysical crossing of bonds can not be excluded. This poses a problem when one is interested in reptation dynamics in melt or ring geometries at any concentration. The modified segmental repulsion model (mSRP) proposed by Sirk et al. [53] offers an easy to implement and computationally cheap way to greatly reduce the number of bond crossing violations. The bond-bond repulsion is modeled analogously to the conservative force in Equation (4) as

(14) $$F_{kl}^{mSRP}=\left\{\begin{array}{cc} b_{rep}\left(1-\frac{d_{kl}}{d_{c}}\right){\hat{d}}_{kl} & \mathrm{if}\;d_{kl}\leq d_{c}\\ 0 & \mathrm{if}\;d_{kl}>d_{c} \end{array}\right.,$$

where $F_{kl}^{mSRP}$ is the bond repulsion force acting between bonds *k* and *l* separated by distance $d_{kl}$ with $b_{rep}$ and $d_{c}$ being the repulsion force constant and bond-bond cutoff distance, respectively. The distance between the two bonds is calculated as the distance between the midpoints of the respective bond vectors. The force acting on the bond decomposes equally into bead forces for beads *i* and *j* in bond *k*, $F_{i}=F_{kl}^{mSRP}\cdot 1/2=F_{j}$. Adjacent bonds are excluded from all segmental repulsion interactions. In order to guarantee the least possible amount of crossing violations but still retain most of the advantages of the standard DPD model some of the simulation parameters need to change when applying the mSRP potential. Namely the bond potential is now modeled as $F_{ij}^{bond}\;=\;b_{ij}\left(b_{0}-r_{ij}\right)r_{ij}$, where $b_{0}=0.91$ is the equilibrium bond distance and the bond force $b_{ij}=50$, $d_{c}=0.8$ and $b_{rep}=25$, and the time step is reduced to Δt=0.01.

## 3. Simulation Details

Linear polymer chains, henceforth called tails and polymer rings, are investigated for various surface coverages σ=E/A, defined as the number of chains ends *E* grafted on the surface divided by the surface area *A*, and polymer lengths *N* reaching from 4 to 256 beads. A ring polymer is defined as a chain with two ends at the surface that are connected via a bond so that $E_{ring}=2E_{tail}$ and therefore for a given σ there are $M_{rings}=M_{tail}/2$ in the system with *M* being the number of polymers. The simulations are carried out with DPD code written by the authors. The size of the simulation box is chosen larger than six times the radius of gyration $R_{g}$ for the investigated species in the *x* and *y* direction. The *z* dimension is selected according to the studied system, e.g., wall with bulk solution on top of the brush or slit brush confinement. The number of chains *M* in the simulation box is determined by the σ of choice we want to investigate as the surface area *A* is set by the aforementioned condition for the *x* and *y* direction. The last parameter we need to establish is the chain length *N* giving us the number of monomer beads $n_{mon}=M\cdot N$ in our simulation. With the given number density $\rho_{DPD}=3$ of our simulation model and the box dimensions $L_{x}$, $L_{y}$, $L_{z}$ determined the total number of DPD beads in the simulation is $n_{tot}=\left(L_{x}L_{y}L_{z}\right)\rho_{DPD}$ and the number of solvent beads therefore is $n_{solv}=n_{tot}-n_{mon}$. For relaxation of the system a $5\times 10^{5}$ step and for data generation a $5\times 10^{6}$ step simulation run with a system sample every 5 steps are performed. Statistical errors are obtained by the block averaging method and are omitted in diagrams if smaller than the symbol size. The investigated properties comprise of density profiles perpendicular to the surface ρ(z), the radius of gyration $R_{g}$, the square root of the squared mean distance of each bead from the polymers center of mass and its components perpendicular $R_{g⊥}$ and parallel $R_{g∥}$ to the wall:

(15) $$\begin{array}{ccc} {\hat{R}}_{g∥}^{2} & = & \frac{1}{2N}\sum_{i=1}^{N}\left[{\left(x_{i}-x_{c.m.}\right)}^{2}+{\left(y_{i}-y_{c.m.}\right)}^{2}\right], \end{array}$$

(16) $$\begin{array}{ccc} {\hat{R}}_{g⊥}^{2} & = & \frac{1}{N}\sum_{i=1}^{N}\left[{\left(z_{i}-z_{c.m.}\right)}^{2}\right], \end{array}$$

(17) $$\begin{array}{ccc} {\hat{R}}_{g}^{2} & = & {\hat{R}}_{g∥}^{2}+{\hat{R}}_{g⊥}^{2}. \end{array}$$

Accordingly, we denote the coordinates of the *i*th monomer as $\left(x_{i},y_{i},z_{i}\right)$ and the corresponding center of mass coordinates of the polymer as $\left(x_{c.m.},y_{c.m.},z_{c.m.}\right)$. In Equations (15)–(17), quantities carrying a hat represent instantaneous values. With 〈…〉 denoting a statistical average, we show in what follows the gyration radius $R_{g}$, as well as its components $R_{g⊥}$ and $R_{g∥}$ defined as $R_{g∥}=\sqrt{\langle {\hat{R}}_{g∥}^{2}\rangle }$; $R_{g⊥}=\sqrt{\langle {\hat{R}}_{g⊥}^{2}\rangle }$; $R_{g}=\sqrt{\langle {\hat{R}}_{g∥}^{2}\rangle +\langle {\hat{R}}_{g⊥}^{2}\rangle }$. We further investigate the shape in form of the asphericity $\delta^{*}$ defined as the degree of being non spherical (i.e., $\delta^{*}=0\to$ sphere, $\delta^{*}=1\to$ rod) and the angle between the normal to the surface and the largest axis of the equivalence ellipsoid $\beta^{*}$ (i.e., the longest eigenvector of the gyration tensor). We study as well a system generated by coating two surfaces with polymer and bringing them into contact. These brushes in slit confinement are investigated as a function of the distance of the surfaces *D*, reaching from the undisturbed brush at infinite separation to the compressed bilayer brush. We take a closer look at the mentioned quantities as well as for the difference of standard DPD and DPD with mSRP bond repulsion added to it. Simulation parameters that are employed are given in the Models and Methods Section 2.

## 4. Results and Discussion

### 4.1. Statistical Properties of the Brush System

**Figure 1:**
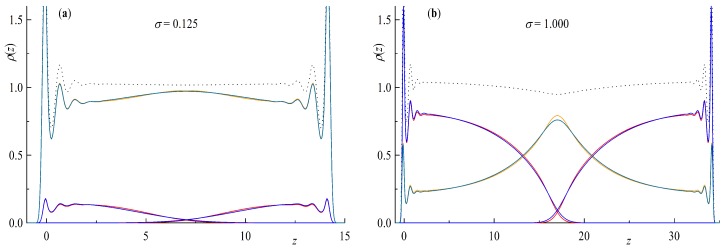
Bead density ρ(z) as a function of distance from surface *z* for a double layer brush system with a surface coverage of (**a**) σ=0.125 brush distance D=14z and (**b**) σ=1.000 and D=34z. Red lines are rings N=64, blue for tails N=32, orange and turquoise for solvent of the ring and linear chain simulations respectively. The dotted line depicts the total bead density of the simulation box.

**Figure 2:**
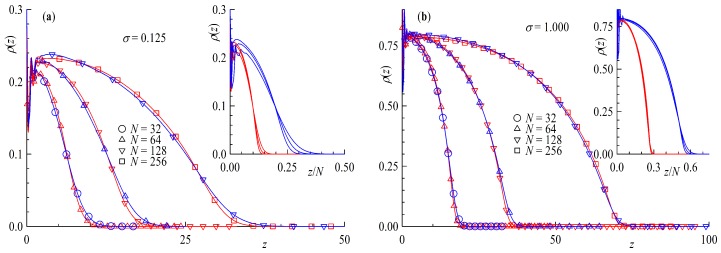
Density profiles ρ(z) as a function of distance from wall *z* for (**a**) surface coverage σ=0.125 and (**b**) σ=1.0. The insets show ρ(z) as a function of the distance scaled by the chain length z/N for the corresponding surface coverage. Red colors are rings and blue for tails.

We start this section with a comprehensive description of the polymer brush system with the main focus on the difference between ring and tail geometries. Firstly we discuss the density profiles along the direction perpendicular to the surface ρ(z) as they are shown in Figure 1 and Figure 2.

Staring with Figure 1 we consider at the monomer densities ρ(z) for two different surface coverages σ. In both cases we can clearly see solvent particles inside the brush layers even for the highest investigated surface coverage σ=1 in Figure 1b. Interestingly, when looking at the total bead density, depicted as the dotted line, we see a dip at D/2=17z in the case of σ=1 only. We can also notice a slightly higher concentration of solvent particles at D/2 for the ring simulations in contrast to the linear ones in both cases although more pronounced for the higher surface coverage perfectly matching the slightly more step like density profiles of the ring polymer brush.

**Figure 3:**
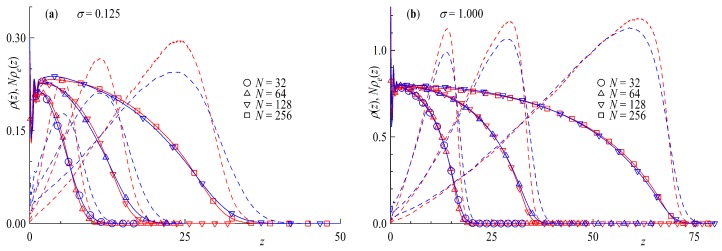
Density profiles ρ(z) and density profiles of end monomers for tails and middle monomers for rings multiplied by chain length $N\rho_{e}\left(z\right)$ as a function of distance from surface *z* for (**a**) surface coverage σ=0.125 and (**b**) σ=1.0$R_{g}$. Chain length *N* is given in different symbols shown. Red colors are rings and blue for tails.

In the following we compare ring and tail brushes in more detail. We study systems with the same amount of effective monomers, e.g., where $N_{ring}=2N_{tail}$ and $M_{ring}=M_{tail}/2$, where the chain length dependence is shown in Figure 2 and marked by different symbols. The surface coverage is defined so that rings count as having two chain ends per molecule and that for any given σ there are always half the number of rings with twice the amount of monomers grafted compared to linear chains $M_{ring}=M_{tail}/2$ and $N_{ring}=2N_{tail}$. Under these conditions it can be clearly seen that the density profiles for rings and tails match quite well. The inset in Figure 2 show the density profiles as a function of the distance scaled by the chain length z/N of the corresponding chain architecture. The density profiles fall onto a master curve with better agreement for the higher surface coverage. The aforementioned factor of 2 in the brush height between ring and tail brushes can be clearly seen. Ring brushes seem to be only very slightly compressed compared to their linear counterparts. The reason for this can be found in Figure 3 where the distribution of end monomers for tails and for middle monomers for rings $\rho_{e}\left(z\right)$ is plotted against the distance from the surface. The distribution shows that the ends (or mid monomers in case of rings, respectively) can be anywhere in the brush, although only few are at the grafting plane. It is interesting to notice that there are slight but systematic differences between rings and tails: for the former the distributions are more sharply peaked, and the tail towards larger *z* is less pronounced. This is not surprising, of course, since the mid monomer is bound by two strands rather than a single one. The presented density profiles and end monomer distributions are in good agreement with the results of Reith et al. and the hard bead spring model found in reference [50]. Our results for the linear chain density profiles conform to the parabolic brush model of Milner, Witten and Cates [17], confirmed also by monomer-based simulations in the case of good solvent [32,33]. Atomistic models for grafted polymer *melts* [39], on the other hand, lead to steplike monomer profiles, akin to that found in Ref. [35] for polymer brushes in solvent of poor quality.

**Figure 4:**
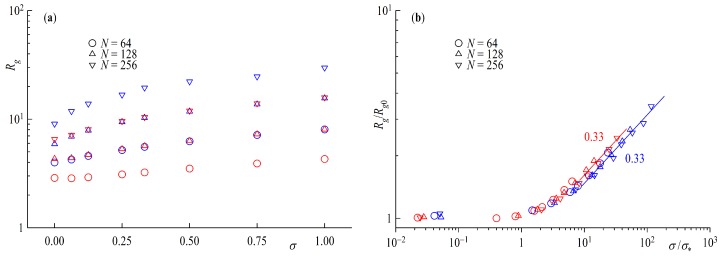
(**a**) Radius of Gyration $R_{g}$ as a function of surface coverage σ and (**b**) $R_{g}$ scaled by a free infinitely diluted chain value $R_{g0}$ as a function of surface overlap concentration $\sigma_{*}$. Straight lines indicate power law fits with scaling exponents shown next to the line. Red symbols are rings and blue for tails.

The radius of gyration $R_{g}$ is shown in Figure 4 as a function of the surface coverage σ. The first feature that stands out in Figure 4a is the similarity of $R_{g}$ for rings and tails with chain lengths following $N_{ring}=2N_{tail}$. As mentioned before rings count as having two ends at the surface per polymer so that the total number of monomers is the same for equal σ and $N_{ring}=2N_{tail}$. This is true especially for surface coverages above the overlap concentration $\sigma_{*}=1/{\left(2\pi R_{g0}\right)}^{2}$, with $R_{g0}$ being the radius of gyration at infinite dilution. The size of the grafted polymers increases with increasing surface coverage as the polymers start to feel each other.

In order to quantify this stretching of the polymers with grafting density, we plot $R_{g}$ normalized by the radius of gyration of an infinitely diluted chain in solution $R_{g0}$ with the same chain length *N* against the surface coverage scaled by the surface overlap concentration in Figure 4b so that the curves for tails and rings fall onto a master curve. Chain statistics for chains below $\sigma_{*}$ are unperturbed as they are without any contact to other polymers, the so called mushroom regime. Starting at $\sigma_{*}$ the increase in size is due to repulsion of the polymers coming into contact with neighboring chains, the beginning of the brush regime. Strongly overlapping polymers exhibit a scaling law $R_{g}\propto \sigma^{\mu }$ with μ=1/3. This scaling is independent of the conformation of the considered system, and in agreement with the Alexander-deGennes blob model [14,15].

**Figure 5:**
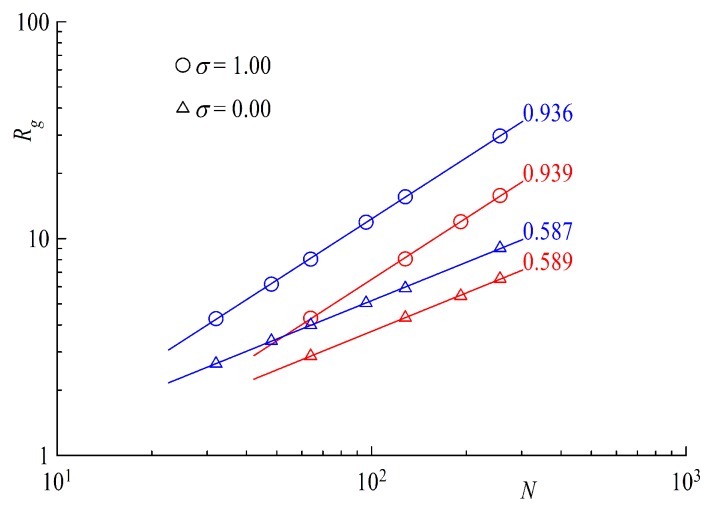
Radius of Gyration $R_{g}$ as a function of chain length *N*. Straight lines indicate power law fits with scaling exponents shown next to the line. Red symbols are rings and blue for tails.

**Figure 6:**
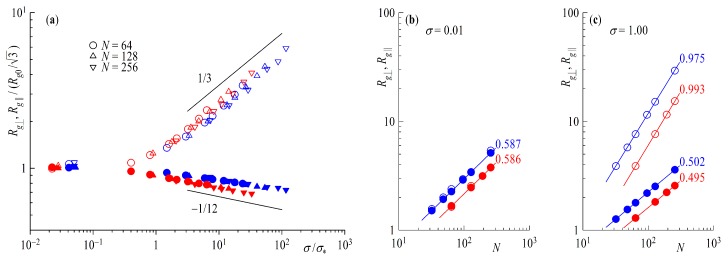
(**a**) Parallel component $R_{g∥}$ (full symbols) and normal component $R_{g⊥}$ (open symbols) of the Radius of Gyration as a function of surface overlap concentration $\sigma_{*}$ and (**b**) as a function of chain length *N* for surface coverage σ=0 (**c**) and σ=1. Straight lines indicate power law fits with scaling exponents shown next to the line. Red symbols are rings and blue for tails.

Looking at the radius of gyration for a single polymer at the surface, e.g., $\sigma \to 0;\sigma \ll \sigma_{*}$ as a function of the chain length, we find that the scaling exponent equals that of a free diluted chain in solution, $R_{g}\propto N^{\nu },\nu =0.588$, as shown by the triangle symbols in Figure 5. A flat surface has no effect on the scaling of a tail or ring in the mushroom regime. On the other hand, if we increase the surface coverage to strongly overlapping chains $\sigma >\sigma_{*}$, as shown by the circle symbols in the same plot, the scaling exponents $R_{g⊥}∼N^{\nu_{⊥}}$ increase strongly up to values of $\nu_{⊥}≅1$, again showing no difference between ring and linear chains. The reason behind the increase is the repulsion of polymers from each other and can be clearly understood if we take a closer look at components of the Radius of gyration, e.g., the parallel component $R_{g∥}$ and normal component $R_{g⊥}$ in Figure 6.

Figure 6a shows the components of the radius of gyration scaled by $R_{g0}/\sqrt{3}$ as a function of the surface density over the overlap concentration $\sigma /\sigma_{*}$. The figure resembles that of Figure 4b as it should. The scaling of the perpendicular component $R_{g⊥}$ with σ retains the form $R_{g⊥}∼\sigma^{\mu_{⊥}}$ the scaling exponent of $\mu_{⊥}≅1/3$ as the major part of the extension of $R_{g}$ stems from expulsion of monomers away from the surface. The parallel component $R_{g∥}$ on the other hand decreases with increasing surface coverage as tails and loops have less and less space, indicating a consistency with a scaling law $R_{g∥}∼\sigma^{\mu_{∥}}$ with $\mu_{∥}≅-1/12$. Looking at the chain length dependence of the $R_{g}$ components from Figure 6 we recover the Flory scaling exponent $R_{g⊥},R_{g∥}∼N^{\nu }$ with ν=0.588 for $\sigma \ll \sigma_{*}$ (Figure 6b), independent of the direction of the component. Repeatedly, a different picture can be seen for $\sigma >\sigma_{*}$ (Figure 6c), where the normal component $R_{g⊥}$ nearly scales with *N* with an exponent $\nu_{⊥}≅1$ and the parallel component $R_{g∥}$ scales with the ideal Gaussian random walk value of $\nu_{∥}=1/2$. The fact that we obtain identical scaling laws for brushes made of chains or rings in a DPD-simulation that allows bond-crossing is not surprising: indeed, concentrated ring polymers, for which bond-crossing is allowed, lack the topological potential that distinguishes them from linear chains. Accordingly, as it has been explicitly confirmed in a recent study of concentrated ring polymer solutions [57], rings without topological restrictions show a scaling of their gyration radius that is identical to that of linear chains. The same also holds true, evidently, for planar brushes. The situation will change as soon as we impose bond-noncorssability, as we will shortly demonstrate.

The above results can be rationalized in the framework of a blob model that envisions the chains/rings as successions of blobs of size $\sigma^{-1/2}$ for $\sigma >\sigma_{*}$. Within each blob, excluded volume interactions are unscreened; accordingly, the number of monomers *g* contained in any such blob scales as:

(18) $$g≅b^{-1/\nu }\sigma^{-1/(2\nu )},$$

with the Flory exponent ν=0.588≅3/5. For a polymer with *N* monomers, this implies that it will consist of $N_{B}=N/g$ such blobs, viz.:

(19) $$N_{B}≅b^{1/\nu }\sigma^{1/(2\nu )}N.$$

As these blobs emerge from the wall on which they are grafted, they grow linearly in the direction perpendicular to the wall, whereas they perform a random walk in the parallel direction. Accordingly, the sizes in the two directions scale as:

(20) $$\begin{array}{ccc} R_{g⊥} & ≅ & \sigma^{-1/2}N_{B} \end{array}$$

(21) $$\begin{array}{ccc} R_{g∥} & ≅ & \sigma^{-1/2}N_{B}^{1/2}. \end{array}$$

Gathering together the above results, we obtain for $\sigma >\sigma_{*}$:

(22) $$\begin{array}{ccc} R_{g⊥} & ≅ & b^{1/\nu }\sigma^{\left(1-\nu )/(2\nu \right)}N \end{array}$$

(23) $$\begin{array}{ccc} R_{g∥} & ≅ & b^{1/(2\nu )}\sigma^{\left(1-2\nu )/(4\nu \right)}N^{1/2}. \end{array}$$

Finally, using $\sigma_{*}≅R_{g0}^{-2}$ together with $R_{g0}≅bN^{\nu }$, the above relations can be recast in the form:

(24) $$\frac{R_{g⊥}}{R_{g0}}≅\left\{\begin{array}{cc} 1 & \mathrm{if}\;\sigma \leq \sigma_{*}\\ {\left(\frac{\sigma }{\sigma_{*}}\right)}^{\left(1-\nu )/(2\nu \right)} & \mathrm{if}\;\sigma >\sigma_{*} \end{array}\right..$$

and

(25) $$\frac{R_{g∥}}{R_{g0}}≅\left\{\begin{array}{cc} 1 & \mathrm{if}\;\sigma \leq \sigma_{*}\\ {\left(\frac{\sigma }{\sigma_{*}}\right)}^{\left(1-2\nu )/(4\nu \right)} & \mathrm{if}\;\sigma >\sigma_{*} \end{array}\right..$$

**Figure 7:**
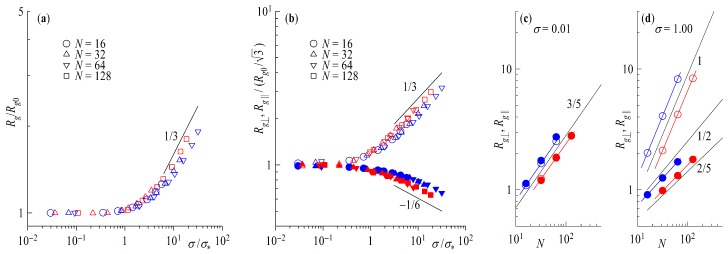
(**a**) Radius of gyration $R_{g}$ scaled by the $R_{g0}$ of infinite dilution as a function of the surface overlap concentration $\sigma /\sigma_{*}$. (**b**) Parallel $R_{g∥}$ (full symbols) and perpendicular $R_{g⊥}$ (empty symbols) component of the radius of gyration scaled by $R_{g0}/\sqrt{3}$ of infinite dilution as a function of the surface overlap concentration $\sigma /\sigma_{*}$. $R_{g∥}$ (full symbols) and $R_{g⊥}$ components (empty symbols) as a function of chain length *N* for (**c**) surface coverage σ=0.01 and for (**d**) σ=1.00. Red symbols are rings and blue for tails. Straight lines indicate power laws with scaling exponents shown next to the line.

Substituting ν=3/5(0.588) in Equations (24) and (25) above, we obtain $\mu_{⊥}=1/3\left(0.35\right)$ and $\mu_{∥}=-1/12\left(-0.075\right)$, as found in the simulation. As mentioned above, the nearly Gaussian scaling for rings in the parallel component is surprising, as it has been previously found [50] that $R_{∥}∼N^{\nu_{∥}}$ with $\nu_{∥}≅2/5$ for non-catenated rings with a topological potential. The advantages of DPD seem to be the limiting factor in this case with its soft potentials and large time steps bond crossing cannot be excluded. To verify this statement, we take a closer look at Figure 7, where we plot results of simulations with an added bond repulsion potential analogous to Figure 4b and Figure 6. Starting with the last plot in Figure 7d we can clearly see the change the additional bond repulsion has on $R_{g∥}$. The ring in the brush now extends laterally to the wall as $R_{g∥}∼N^{\nu_{∥}}$ with $\nu_{∥}≅2/5$, whereas $R_{g⊥}$ retains its scaling of $\nu_{⊥}≅1$. For $\sigma \ll \sigma_{*}$ in Figure 7c we also find that the scaling of $R_{g⊥};R_{g∥}\propto N^{\nu }$ with ν=3/5 is maintained, as the topological repulsion between neighboring rings plays no role at the mushroom regime. As far as the scaling with surface coverage for $\sigma >\sigma_{*}$ is concerned, The scaling of the perpendicular component $R_{g⊥}$ retains its characteristic scaling exponent, $R_{g⊥}\propto \sigma^{\mu_{⊥}}$ with $\mu_{⊥}≅1/3$, as seen in Figure 7a,b; the topological repulsion plays no role in the directed motion of the blobs perpendicular to the wall. On the other hand, the fact that the blobs no longer perform a random walk parallel to the wall affects the exponent $\mu_{∥}$, as we now find $R_{g∥}∼\sigma^{\mu_{∥}}$, with the exponent now changed by a factor 2, from $\mu_{∥}=-1/12$ to $\mu_{∥}=-1/6$. This change of the exponent as a consequence of topological constraints is strongly reminiscent of the situation for concentrated ring polymer solutions, where in the scaling law of the gyration radius with concentration, $R_{g}/R_{g,0}∼{\left(c/c_{*}\right)}^{x}$ for concentrations *c* exceeding the overlap value $c_{*}$, the exponent *x* changes from x=−1/8 without topological interactions to double this value, x=−1/4, when the latter are taken into account [57].

**Figure 8:**
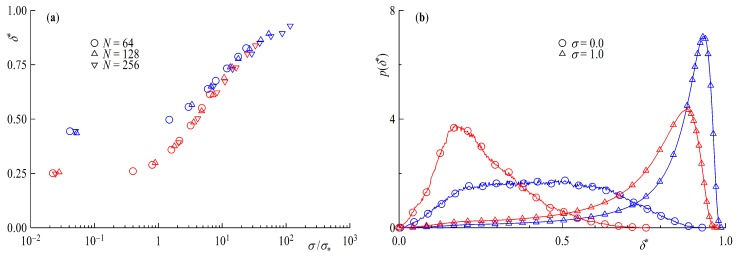
(**a**) Asphericity $\delta^{*}$ as a function of the surface overlap concentration $\sigma /\sigma_{*}$ and (**b**) asphericity frequency distribution $F(\delta^{*})$ for $N_{R}=128$ and $N_{L}=64$. Red symbols are rings and blue for tails.

Next we take a look at the asphericity $\delta^{*}$ and the perpendicularity $\beta^{*}$. Starting with Figure 8a, for surface coverages below the overlap concentration $\delta^{*}$ coincides very well with the predicted values of $\delta_{R}^{*}=0.2551$ for rings and $\delta_{L}^{*}=0.4303$ linear chains in solution [58], with rings being the more spherical species. With increasing surface coverage starting at the overlap, concentration rings and tail geometries are getting compressed to a more rod-like shape approaching the limiting value of 1 of the completely stretched form. Taking a look at the distribution of $\delta^{*}$ in Figure 8b, one can see the difference between rings an linear chains are even more pronounced. For $\sigma <\sigma_{*}$ the distribution for rings is more sharply peaked around $\delta^{*}=1/4$ while its linear counterpart is flat and broad. For σ=1, on the other hand, the histogram for the ring geometry shows a more pronounced tail in direction of a more spherical shape. This might be another contributing factor for the slightly more step-like density profiles of ring brushes.

**Figure 9:**
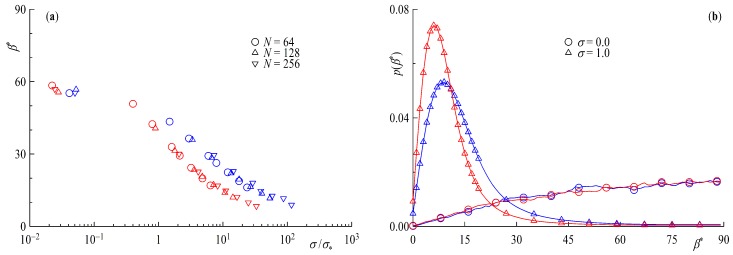
(**a**) Angle between the normal to the surface and the largest eigenvector of the gyration tensor $\beta^{*}$ as a function of the surface overlap concentration $\sigma /\sigma_{*}$ and (**b**) frequency distribution $F(\beta^{*})$ for $N_{R}=128$ and $N_{L}=64$. Red symbols are rings and blue for tails.

To complete the investigation of the statistical properties of the ring and linear tail brush we take a closer look at the angle between the normal to the surface and the largest eigenvector of the gyration tensor, i.e., the largest axis of the equivalence ellipsoid. Analogous to the asphericity we plot $\beta^{*}$ as a function of the surface overlap concentration Figure 9a and the distribution $p(\beta^{*})$ for two different limiting surface coverages σ=0 and σ=1 in Figure 9b. In the case of low surface coverage, a relatively large angle of around $60^{\circ }$ is observed, implying a more surface parallel shape with virtually no difference between ring and tail polymers. Taking a closer look at the distribution for σ=0, however, it becomes clear that there is no real preference of any one angle, as the peak is broad and flat, although larger angles are more probable and again no distinction due to the different geometries is perceivable. For high surface coverages well above the overlap concentration the resulting angles are getting progressively smaller, i.e., more perpendicular. The discrepancy between the different geometries is very small, only when we look at the distributions can we recognize a difference. The peak for the ring brush is sharper than that of the linear chains. We note, further, that the distribution $p(\beta^{*})$ bears significant similarities with its counterpart for short, rigid-chain brushes, as established in atomistic simulations [43].

### 4.2. Decreasing the Distance between Two Brushes

**Figure 10:**
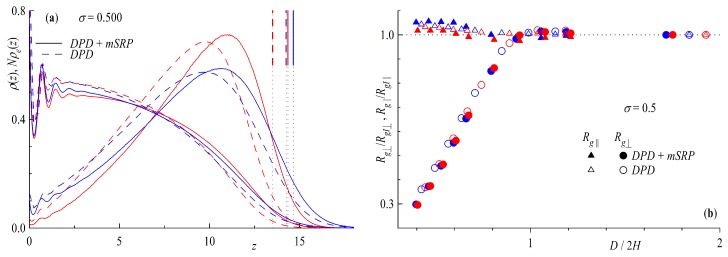
(**a**) Density profiles ρ(z) and end (mid) monomer distributions $\rho_{e}\left(z\right)$. The short strait lines vertical lines symbolize the 2.5% brush height criteria (see text). (**b**) Parallel $R_{g∥}$ and perpendicular component $R_{g⊥}$ of the radius of gyration scaled with value of infinite separation $R_{gI∥}$ and $R_{gI⊥}$. Both as a function of brush distance *D* scaled by two times the brush height at infinite separation *H* at a surface coverage of σ=0.5. Red symbols are rings with $N_{rings}=64$ and blue for tails $N_{tails}=32$. Dashed lines and empty symbols for standard Dissipative Particle Dynamics (DPD) and full lines and symbols for DPD + modified Segmental Repulsion Potential (mSRP) (with bond repulsion potential).

In this section we take a closer look at what happens to our brush if we put it in contact with a twin. We focus our interest at a single chain length and surface coverage for each geometry, i.e., $N_{ring}=64$ and $N_{tail}=32$ both at σ=0.5 corresponding to $\approx 26\sigma_{*}$ for rings and $\approx 22\sigma_{*}$ for linear chains, chosen for the similarity of the ring and tail brush. Included in our comparisons will be data which was generated with the addition of the bond repulsion potential discussed in Section 2.3. We investigate the properties as a function of the interbrush distance *D* scaled by the height of the unperturbed brush *H* at infinite separation, defined as the height at which the monomer density ρ(z) of the brush decreases to 2.5%, as seen in the short vertical lines at the top right corner of Figure 10a.

Regarding the density profiles in Figure 10a, we find a more elongated brush for the simulations including the bond repulsion potential (full lines) compared to their standard DPD counterparts (dashed lines) coinciding with an decrease in density closer to the surface. Comparing ring and tail geometries, we find an even more step-like density profile for rings as that already seen in Figure 3. The end monomer (linear chains) and mid monomer (rings) distributions multiplied by the chain length are plotted as well, displaying a shift to larger distanced from the surface (swelling of the brush). The brush height, defined above, increases in accordance with the shift in end (mid) monomer distributions shown, decreasing the difference of ring and tail brushes but retaining the fact of higher linear brushes.

Plotted in Figure 10b are the normal $R_{g⊥}$ and parallel $R_{g∥}$ component of the radius of gyration as a function of brush distance scaled by brush height. From the point of contact, i.e., D/2H=1, to closer confinements, a drastic decrease of the perpendicular component occurs, and is slightly more pronounced for ring geometries for both DPD and DPD + mSRP. The effect is larger for bond repulsion simulations but followsthe same general trend. Interestingly, the parallel component increases more profoundly for the linear chains, with the standard DPD data lying in between rings and tail geometries. This leads to an overall larger decrease in $R_{g}$ for rings, leading to a more compact form.

**Figure 11:**
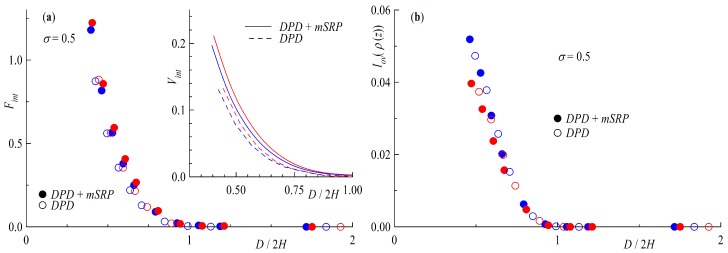
(**a**) Interaction force $F_{int}=\int_{-\infty }^{0}\rho \left(z\right)zddz\cdot \left(-1\right)$ generated by polymer beads acting on the walls of the system. The insert depicts the interaction potential $V_{int}=\int F_{int}$. (**b**) Integral of overlap $I_{ov}\left(\rho \left(z\right)\right)=\int_{0}^{D}\rho_{1}\left(z\right)\rho_{2}\left(z\right)dz$. Both plots are shown as a function of brush distance *D* scaled by two times the brush height at infinite separation *H* at a surface coverage of σ=0.5. Red symbols are rings with $N_{R}=64$ and blue for tails $N_{L}=32$.

With decreasing distance of the polymer coated surfaces the grafted chains and rings start to increase their interactions with the wall and each other due to the compression of the brush. The results of this effect are shown in Figure 11a with the interaction force $F_{int}=\int_{-\infty }^{0}\rho \left(z\right)zddz\cdot \left(-1\right)$, the integral of the monomer density behind the wall multiplied by the wall repulsion *d* at the distance *z* (see Section 2.2 for details on wall repulsion). The interpenetration of brushes into each other is plotted in Figure 11b as the integral of overlap $I_{ov}\left[\rho \left(z\right)\right]=\int_{0}^{D}\rho_{1}\left(z\right)\rho_{2}\left(z\right)dz$, with $\rho_{1}\left(z\right)$ and $\rho_{2}\left(z\right)$ being the monomer densities of brush 1 and 2, respectively. Starting with Figure 11a an increase in monomer density behind the wall with decreasing distance *D* of the brushes is found for all systems. In both cases the increase for rings is larger than for linear chains, being more distinct in the case of added bond repulsion. This can also be seen in the insert of Figure 11a showing the interaction potential $V_{int}$, the integral of the interaction force as a function of scaled brush distance. Even without the bond repulsion potential ring brushes are effectively more repulsive than their linear counterparts, with an increased effect for added segmental repulsion. The reason behind this can be understood if we take a look at the data of Figure 11b. Comparing ring and tail geometries, rings always show a smaller overlap than their linear counterparts. This effect is not only due to the bond repulsion potential, although it is increased by it. Linear chains, with their free chain ends, more easily penetrate the other brush, whereas rings are more strongly repelled to a more compact state, increasing the force on the wall in the process.

## 5. Conclusions

We have presented results on the conformations and interactions of polymer brushes employing DPD simulations, putting particular emphasis on a systematic comparison between brushes of linear and ring polymers, the latter being in this case chains that are grafted on the walls on both their ends. We have quantitatively obtained all the features of the high grafting-density regime, and in particular the scaling of the perpendicular and lateral extensions of the polymers both with the degree of polymerization and the grafting density. A key finding of our work is that in the absence of consideration for the topological interaction between rings, i.e., neglecting the prohibition of bond-crossing, rings and chains become indistinguishable; on the contrary, when the topological interactions are properly taken into account, ring brushes become distinct from their linear counterparts: the lateral conformation of the rings is no longer a random walk, since the topological interaction cannot get screened out at any monomer concentration; concomitantly, the scaling of $R_{g∥}$ with both the degree of polymerization and the grafting density picks up exponents that are unique to rings and carries the signature of ring conformations that form a class of their own: they are neither ideal walks nor self-avoiding walks, a feature already observed in bulk ring polymer solutions. The effects of the topological constraints are also manifest in the interbrush interaction, where we have established that the ring brushes feature a more repulsive interaction potential than their linear counterparts, implying that ring polymers, when grafted onto colloids, will be stronger agents against colloidal coagulation than linear ones [59]. This is an intriguing counterpoint to the case of added *non-grafted, non-adsorbing* polymer, where it has been recently found that free rings in solution lead to stronger depletion attractions between colloids than free linear chains. Future work with DPD simulations, which have the great advantage of including explicit solvent in an economic way, will focus on studying their properties under shear and the resulting effects on friction and lubrication.

## Abbreviations

The following abbreviations are used in this manuscript:

DPD	Dissipative Particle Dynamics
mSRP	modified Segmental Repulsion Potential
